# Effect of Volatile Organic Compounds Adsorption on 3D-Printed PEGDA:PEDOT for Long-Term Monitoring Devices

**DOI:** 10.3390/nano11010094

**Published:** 2021-01-04

**Authors:** Giorgio Scordo, Valentina Bertana, Alberto Ballesio, Rocco Carcione, Simone Luigi Marasso, Matteo Cocuzza, Candido Fabrizio Pirri, Matteo Manachino, Manuel Gomez Gomez, Alessandra Vitale, Angelica Chiodoni, Emanuela Tamburri, Luciano Scaltrito

**Affiliations:** 1 Chilab-Materials and Microsystems Laboratory, Department of Applied Science and Technology (DISAT), Politecnico di Torino-Via Lungo Piazza d’Armi 6, 10034 Chivasso (TO), Italy; simone.marasso@polito.it (S.L.M.); matteo.cocuzza@infm.polito.it (M.C.); fabrizio.pirri@polito.it (C.F.P.); matteo.manachino@polito.it (M.M.); manuel.gomezgomez@polito.it (M.G.G.); luciano.scaltrito@polito.it (L.S.); 2 Department of Chemical Sciences and Technologies, Università degli Studi di Roma, “Tor Vergata”, Via della Ricerca Scientifica, 00133 Rome, Italy; rocco.carcione@uniroma2.it; 3 CNR-IMEM, Parco Area delle Scienze, 37a, 43124 Parma, Italy; 4 Department of Applied Science and Technology (DISAT), Politecnico di Torino, C.so Duca degli Abruzzi 24, 10129 Turin, Italy; alessandra.vitale@polito.it; 5 Center for Sustainable Future Technologies, Istituto Italiano di Tecnologia, via Livorno 60, 10144 Turin, Italy; Angelica.Chiodoni@iit.it

**Keywords:** PEGDA, PEDOT, additive manufacturing, stereolithography, cumulative adsorption

## Abstract

We report on the preparation and stereolithographic 3D printing of a resin based on the composite between a poly(ethylene glycol) diacrylate (PEGDA) host matrix and a poly(3,4-ethylenedioxythiophene)-poly(styrenesulfonate) (PEDOT:PSS) filler, and the related cumulative volatile organic compounds’ (VOCs) adsorbent properties. The control of all the steps for resin preparation and printing through morphological (SEM), structural (Raman spectroscopy) and functional (I/V measurements) characterizations allowed us to obtain conductive 3D objects of complex and reproducible geometry. These systems can interact with chemical vapors in the long term by providing a consistent and detectable variation of their structural and conductive characteristics. The materials and the manufacture protocol here reported thus propose an innovative and versatile technology for VOCs monitoring systems based on cumulative adsorption effects.

## 1. Introduction

The always-growing number of morbidity events due to environmental pollution has led to an increased awareness of the main sources of exposure that could seriously affect human health. This problem especially regards indoor pollution, which is strongly related to the ever-improving isolation of the places where people live, and thus is of big concern in developed countries [1]. Volatile organic compounds (VOCs) are an example of indoor pollutants, since they are gases emitted from solids or liquids with low boiling points (e.g., solvents used in varnishes). Although the monitoring of VOCs is mainly performed by electrochemical sensors [2,3], a new class of devices based on cumulative adsorption effects [4,5] has recently been attracting more and more attention in the scientific community.

Cumulative measuring devices have been reported to be suitable for monitoring the presence of organic solvents [6,7,8,9]. There are several types of working principle of such devices, which are related to the changes of their optical, redox, or electrical properties when exposed to chemical vapors. In particular, the measurement of their conductivity variation provides several advantages, such as good sensitivity, simple measurement setup (an ohmmeter is enough to collect the data) and easiness of integration with other systems [10].

In this context, conductive polymers (CPs) are an interesting class of low-cost adsorption materials due to their peculiar optical, electrochemical and charge transport properties, and the versatility of their preparation methods [11,12,13,14,15]. Therefore, the exploitation of CPs in cumulative measuring devices can represent a promising solution to overcome the limitation of oxide-based cumulative sensors requiring high power consumption for preparation and operation [16]. As a consequence, during the last ten years many efforts [17,18,19] have been made for the integration of low-cost CPs into electronic devices to obtain cumulative measuring systems.

Among the various CPs, such as polyaniline (PANI), Polyfuran (PFu), polypyrrole (PPy) and polythiophenes (PTs), poly(3,4-ethylene-dioxythiophene) (PEDOT) has already found application in different fields [20], due to its high chemical stability and conductivity. Several researchers have demonstrated that the incorporation of PEDOT into Polyacrylamide (PAM) [21], Polyethylene terephthalate (PET) [22] or Polyvinyl alcohol (PVA) [23] matrices allows for the production of conductive materials suitable for the detection of nitrogen dioxide [24], ammonia [25], acetone, ethanol, methanol and tetrahydrofuran vapors at room temperature [26]. From this perspective, the coupling of PEDOT with a 3D printable polymer can be considered a reliable manufacturing strategy for producing cumulative adsorption materials with customized architectures.

Three-dimensional printing, or additive manufacturing, has been largely employed for producing polymer-based materials for biomedical applications, soft electronics, and sensors [27]. However, few works have been reported on the 3D printing of a poly(ethylene glycol) diacrylate (PEGDA) and poly(3,4-ethylenedioxythiophene)-poly(styrenesulfonate) (PEDOT:PSS) blend [28,29]. Furthermore, the preparation of conductive systems for the assembly of monitoring devices has essentially been restricted to the extrusion-based printing method [30,31].

In this context, the aim of this work is the fabrication, by stereolithography (SL) 3D printing, of PEGDA:PEDOT conductive composite systems as possible active materials for VOCs monitoring devices. Specifically, a purification treatment was employed to enhance the charge transport properties of commercial PEDOT:PSS. Then, blends obtained by mixing different amounts of PEGDA and PEDOT:PSS polymers were used for printing 3D double helical-shaped structures by means of a customized SL printer. The morphological and structural features of the printed objects were evaluated by FESEM and Raman spectroscopy analyses. The VOCs monitoring capabilities of the produced samples were assessed by analyzing the electrical response modulated by vapor adsorption after exposure to two common organic solvents, i.e., acetone and ethanol, through current/voltage (*I*/*V*) measurements. The variation in the electrical conductivity with the duration of the exposure to vapors was related to the conformational changes induced in the PEDOT phase. In addition, the effects produced by vapors on the structural features of the entire polymer phase were evaluated by means of Raman spectroscopy analysis.

The obtained achievements show how a controlled integration of the conductive PEDOT:PSS material into a PEGDA matrix offers the opportunity to print 3D complex structures that can be reliably applied in the gas monitoring field by using the cumulative adsorption effect.

## 2. Materials and Methods

### 2.1. PEDOT:PSS Treatment

The poly(3,4-ethylenedioxythiophene)–poly(styrenesulfonate) (PEDOT:PSS) filler was prepared from a commercial solution. Namely, Clevios™ PH1000, from now on referred to as pristine PEDOT:PSS, was dispersed in a 0.5 M H_2_SO_4_ solution in a 1:10 ratio, and purified as previously reported [32]. The PEDOT:PSS-based powders thus obtained were dispersed in ethanol, and the aggregates eventually formed were reduced by using an Ultra-Turrax^®^ (IKA, model T25, impeller 10 G, Staufen, Germany) homogenizer for 15 min at 30,000 rpm. The ethanol excess was separated and removed by centrifugation at 5000 rpm for 10 min. The sample accordingly produced was named treated PEDOT:PSS.

### 2.2. Stereolithography Resin Preparation

The SL resin was prepared by using photocurable poly(ethylene glycol) diacrylate (PEGDA) as the host matrix. PEGDA with an average molecular weight Mn ~575 was purchased from Millipore Sigma (Burlington, MA, USA). The bis(2,4,6-trimethylbenzoyl)-phenylphosphineoxide (Irgacure 819) radical photoinitiator and dimethyl sulfoxide (DMSO) were purchased from Millipore Sigma (Burlington, MA, USA).

Briefly, the radical photoinitiator (1 wt. % with respect to PEGDA) was initially dissolved in PEGDA by sonication at 30 kHz (Branson, Digital Sonifier SFX250, Danbury, Connecticut, USA) for 10 min. Then, treated PEDOT:PSS was mixed with PEGDA and DMSO (5 wt. % with respect to the total resin) under magnetic stirring at 600 rpm for 15 min. Different weight ratios between matrix and filler were investigated. In detail, 5 wt. %, 10 wt. %, 15 wt. %, 25 wt. %, 35 wt. % and 45 wt. % of treated PEDOT:PSS were added to the PEGDA with respect to the total resin weight. FT-IR spectroscopy (Nicolet iS50, Thermo Fisher, Madison, Wisconsin, USA) was employed for investigating the UV curing conversion during the SL printing of the resin, hereafter simply referred to as PEGDA:PEDOT.

In order to achieve a full exhausting of any residual initiator and an improvement of the physical–chemical properties and stability of the material, a post-curing step of the 3D-printed parts was performed at 7 mW/cm^2^ for 10 min. The resin conversion before and after post-curing was then evaluated.

### 2.3. D Printing of PEGDA:PEDOT

A customized SL printer (Microla Optoelectronics s.r.l., Chivasso, Italy) [33] was employed for sample fabrication. It builds up objects following a top-down layer-by-layer approach exploiting light curing with a 405 nm wavelength laser. A previous work reported the optimal printing parameters for PEGDA:PEDOT-based resins: 10 mW laser nominal output power and 2000 mm·s^−1^ laser scan velocity. At the end of the printing process, each sample was carefully rinsed with isopropyl alcohol and dried under compressed air. The post-curing treatment was performed under UV light (Hamamatsu LC8 lamp, Shizuoka, Japan) in nitrogen atmosphere for 20 min.

As regards Raman spectroscopy and the photocuring conversion, 3D-printed samples (5 mm × 5 mm × 10 mm) were analyzed for each different condition.

**Figure 1:**
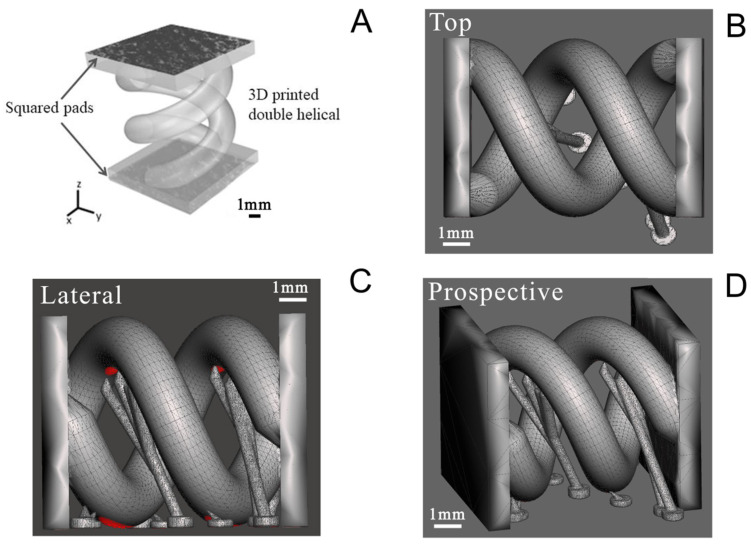
Computer-aided design drawing of double helix structure. (**A**) General view showing the pads for contact during electrical characterization; (**B**) top view; (**C**) lateral view; and (**D**) prospective view.

A double helical structure was finally printed with the PEGDA:PEDOT resin with the optimized treated PEDOT:PSS (Figure 1A). The geometry was designed to enhance the PEGDA:PEDOT’s exposed surface in a tight volume and indirectly to demonstrate the possibility of printing complex-geometry objects with the optimized conductive composite resin. The double helix was designed as two rods following a 360° turn along an 8 mm height, with each rod having a circular section of 2 mm diameter (Figure 1B), and adding some tiny temporary support structures that are shown in Figure 1C,D. The structure was also equipped with two squared pads at its extremities, both to fix in place the two rods and to allow for an easier contact during electrical characterization.

### 2.4. Morphological and Structural Characterization

#### 2.4.1. FESEM and Raman Spectroscopy Analyses

The morphological features of the treated PEDOT:PSS and printed PEGDA:PEDOT were investigated by ZEISS Merlin and Supra 40 scanning electron microscopes (Obekochen, Germany).

The molecular structures of the treated PEDOT:PSS and printed PEGDA:PEDOT before and after organic solvent vapors exposure were evaluated by Raman spectroscopy by a Renishaw inVia Raman microscope (Renishaw plc, Wotton-under-Edge, UK) equipped with a Leica DMLM microscope with a 50× objective. For the excitation, a 514.5 nm Ar laser with an output power of 50 mW (5 mW at the sample) was used. All spectra were recorded using 5 accumulations (20 s exposure) and deconvolved with Lorentian line functions.

#### 2.4.2. FT-IR Analysis

The PEGDA:PEDOT resin photocuring conversion was checked by Fourier transform infrared (FT-IR) spectroscopy, using a Thermo Scientific™ Nicolet™ iS50 spectrometer (Madison, WI, USA) in Attenuated Total Reflectance (ATR) mode. To this aim, the decreasing of the absorption band area related to the reactive functionality (C=C acrylic group of PEGDA centered at 1635 cm^−1^) was monitored. Specifically, the conversion yield was calculated from the ratios between the areas of this band and those at 1720 cm^−1^, assigned to the C=O carbonyl group, before and after polymerization, as reported in Equation (1):

(1) $$Conversion\;\left(\%\right)=\left(1-\frac{A/A_{ref}}{A_{0}/A_{0,\;ref}}\right)\cdot 100$$

where *A*_0_ and *A* are the area of the absorption band of the reactive functionality before and after curing, respectively, while *A*_0,*ref*_ and *A_ref_* are the areas of the C=O peak before and after curing, respectively. FT-IR analyses were at least performed on three different spots of a sample under the same conditions to confirm the reproducibility of the results. Finally, the average conversion value and the standard deviation were calculated and reported.

### 2.5. VOCs Exposure Experiments

The experimental set-up consisted in a sealed chamber that, before each experiment, was washed with deionized water and then flushed with clean air to reduce the contamination. The squared pads of the helical samples were covered with silver conductive paste to establish a good ohmic contact with the testing probes [34]. Acetone and ethanol were the investigated organic solvents. Briefly, after a printed sample was exposed to a solvent-saturated atmosphere for different periods (10 min, 20 min, 30 min, 60 min and 120 min), it was dried at room temperature before Raman spectroscopy and conductivity measurements. The conductivity measurements were performed using a potentio-dynamic current/voltage (*I*/*V*) Keithley 6430 instrument applying a linear voltage between −1 and 1 V. From the resistance values and the sample geometry, the conductivity was calculated and expressed in S·cm^−1^. The influence of the exposure and drying times was investigated and correlated to all the obtained results.

## 3. Results

### 3.1. Morphology and Structure of PEGDA:PEDOT

**Figure 2:**
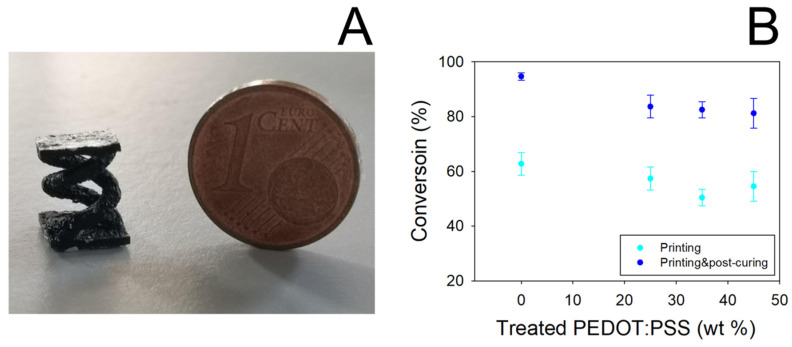
(**A**) 3D-printed double helical sample from PEGDA:PEDOT resin (printing parameters 50 mW laser power, 2000 mm s^−1^ scan velocity). (**B**) Conversion yield of PEGDA as a function of treated PEDOT:PSS concentration. Data were obtained by FT-IR spectroscopy analysis on 3D-printed samples before and after the post-curing process.

In Figure 2A a picture showing a typical double helical object obtained by SL printing PEGDA:PEDOT resin is reported. It can be noted that the presence of the treated PEDOT:PSS filler does not affect the photocuring ability of the PEGDA matrix to assume defined 3D geometries. Nevertheless, the introduction of the filler slightly reduced the PEGDA conversion, as shown in Figure 2B.

Even if a 10% decrease in the conversion yield of PEGDA is obtained, in any case it is observed that the yield remains constant by varying the filler loading from 25 to 45% wt. Moreover, conversion values higher than 80% are obtained when the samples are subjected to the post-curing process, thus highlighting the essential role of this processing step in producing reliable and manageable objects.

**Figure 3:**
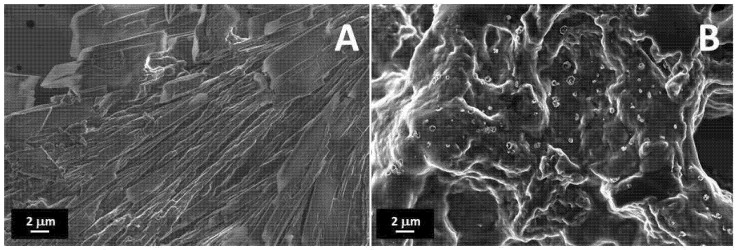
FESEM images of (**A**) treated PEDOT:PSS, and a (**B**) PEGDA:PEDOT printed double helical structure with a 45 wt. % filler inclusion.

FESEM images of the treated PEDOT:PSS and printed PEGDA:PEDOT with a 45% wt. filler inclusion are reported in Figure 3A,B, respectively. It is worth observing that surface metallization was not necessary for the SEM analysis to point out the conductive properties of both samples.

As one can note in Figure 3A, the treated PEDOT:PSS is composed of columnar structures, reasonably attributable to the rearrangement of PEDOT:PSS into nanofibrils after H_2_SO_4_ treatment [35]. The protons released from acid are in fact expected to neutralize some PSS ions to PSSH, causing the weakening of coulombic attraction with PEDOT moieties [36]. In this sense, hydrogen sulfate ions replace PSS ions as counter ions inducing a phase segregation between PEDOT and PSS units and a rearrangement of the polyelectrolyte chains in linearly oriented structures.

The observation of the surface morphology of the printed object with the higher filler loading (Figure 3B) confirms the formation of a composite resin with a homogenous globular morphology without significant phase separation between the PEGDA and treated PEDOT:PSS components. This means that, although high filler loading levels are adopted, an efficient mixing between the two materials can still be obtained at the micro scale while maintaining a high conversion yield of the photocurable PEGDA polymer.

**Figure 4:**
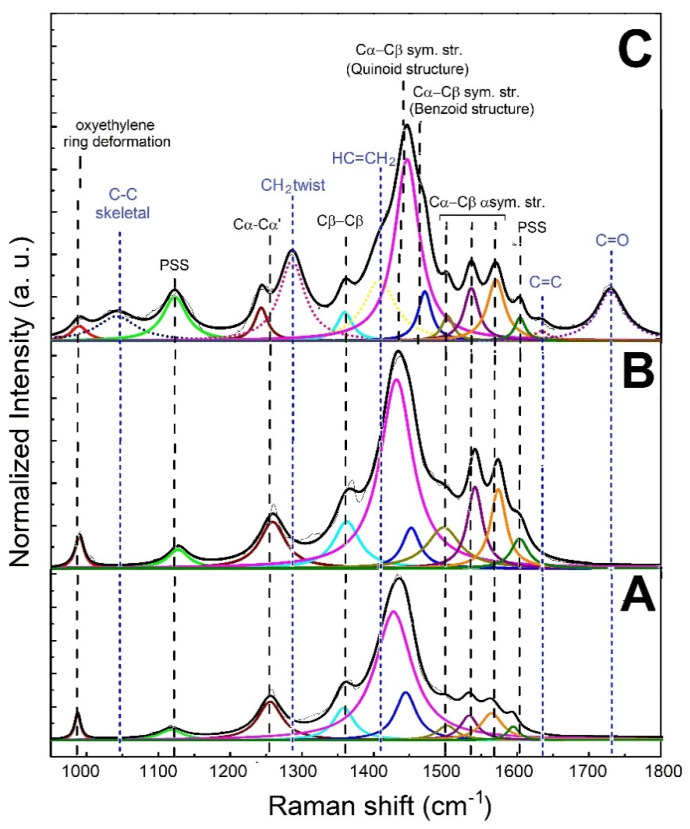
Raman spectra of (**A**) untreated PEDOT:PSS, (**B**) treated PEDOT:PSS and (**C**) printed PEGDA:PEDOT.

The analysis of the structural features of filler and composite was conducted through Raman spectroscopy. The Raman spectra, along with peak designations of the untreated PEDOT:PSS, treated PEDOT:PSS and printed PEGDA:PEDOT with a 45 wt. % filler inclusion, are shown in Figure 4A–C, respectively. The spectra were acquired in the range between 950 and 1800 cm^−1^, and were normalized to the predominant feature at about 1440 cm^−1^.

As shown in Figure 4A,B, except for the features related to PSS at 1120 and 1605 cm^−1^, all the peaks in the spectrum of treated PEDOT:PSS can be attributed to the PEDOT main chain. On the other hand, in addition to the signals related to the PEDOT:PSS phase, the Raman spectrum of the printed PEGDA:PEDOT exhibits additional peaks (blue dotted lines) attributable to the PEGDA chains (Figure 4B). This result further confirms an effective mixing between matrix and filler.

An in-depth analysis of the spectral bands offers more detailed information on the molecular structure of the main components. In particular, the features at about 1440 and 1460 cm^−1^ can be assigned to the C_α_−C_β_ and C_α_ = C_β_ symmetric stretching modes, respectively related to the coil-like benzoid and quinoid arrangements typically found in PEDOT systems [37,38,39,40]. Specifically, an extended quinoid conformation enables a closer packing of PEDOT chains [41,42], in which the π-electron delocalization is facilitated and a noticeable charge transport can be achieved. The percentage content of the extended quinoid structure (% quinoid) can be determined by using Equation (2):

(2) $$\%\;\mathrm{quinoid}=\frac{I_{qui}}{I_{qui}+I_{benz}}\cdot 100$$

where *I_qui_* and *I_benz_* are the Raman integrated intensity values of the quinoid C_α_−C_β_ and benzoid C_α_=C_β_ symmetric stretching modes, respectively.

% quinoid values of 90 and 88% are obtained for treated PEDOT:PSS and printed PEGDA:PEDOT. Considering that the % quinoid value of the untreated PEDOT:PSS sample is 80%, these results suggest that the acidic treatment to which the commercial PEDOT:PSS was subjected before insertion into the PEGDA matrix was successful in inducing a linear conformation in the PEDOT:PSS adducts by preserving the PEDOT conducting oxidized state. This behavior is consistent with previous literature [43,44]. This molecular arrangement is also maintained in the composite, highlighting how the mixing does not disturb the conformation of PEDOT chains but favors the setting-up of ordered PEDOT:PSS regions available for charge transport in the composite material. In particular, the decrease in the FWHM value of the quinoid peak from 63 to 55 to 31 cm^−1^, for untreated PEDOT:PSS, treated PEDOT:PSS and printed PEGDA:PEDOT samples, suggests an increase in the crystalline order, plausibly ascribed to the alignment of PEDOT crystalline domains in a linear conformation [45].

The broad bands related to the PEGDA phase suggest a poor structural ordering of the chains with the formation of a mostly amorphous system. However, a value of 5 for the ratio between the integrated intensity of the peaks related to the C=O and C=C stretching vibrations highlights the significant crosslinking of acrylate [46]. This result is in accordance with a Young’s module value of around 21 MPa previously measured for other PEGDA:PEDOT-based materials [32].

### 3.2. Conductivity of PEGDA:PEDOT

**Figure 5:**
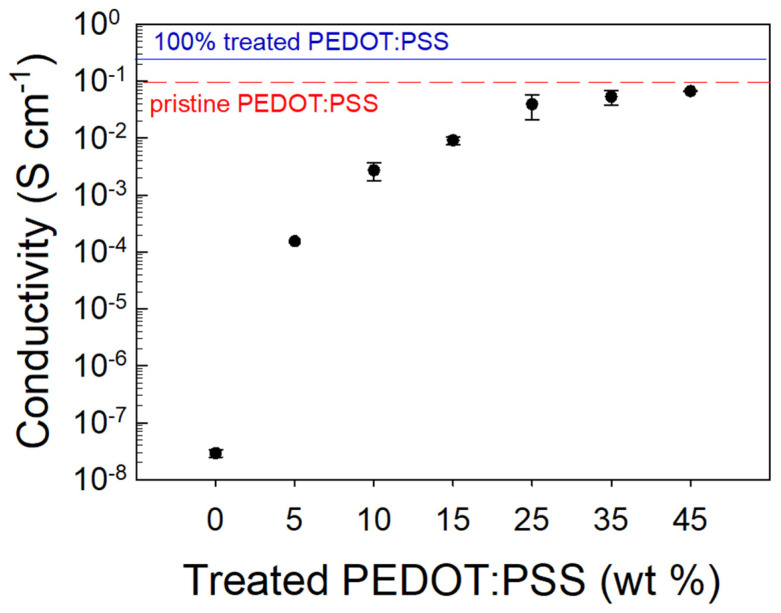
Conductivity of the PEGDA:PEDOT samples at different treated PEDOT:PSS contents.

All the samples printed from resins with different filler loadings were subjected to I/V measurements to evaluate their conductive properties. In Figure 5, the electrical conductivity of the various samples is reported as a function of treated PEDOT:PSS percentage content in the PEGDA matrix.

We can note that the electrical conductivity variation vs. the filler loading follows a sigmoidal curve. Specifically, for small treated PEDOT:PSS contents, a high resistivity of the composite is detected, but by increasing the conductive filler fraction, an enhancement of conductivity with an asymptotic trend is found. In particular, it is observed that the onset of charge transport features occurs at a ∼25 wt. % loading, providing a composite conductivity of 0.05 S·cm^−1^. This result is consistent with the percolation threshold (v_c_) value that can be estimated from a differential analysis of the fitting curve (R^2^ = 0.95, v_c_ = 0.25). Such a high threshold value, if compared to the typical values ranging between 0.02 and 0.05% [47,48] commonly found in the literature, can be reasonably explained by considering the size and shape of the filler used in this study. The highest conductivity is achieved for those samples with a 45 wt. % treated PEDOT:PSS inclusion providing a σ≈ 0.055 S·cm^−1^. For filler percentages above 45 wt. %, the resin printability was lost due to the high viscosity of the composite material. Indeed, this viscosity did not allow for a homogeneous recoating and spreading of the liquid resin on the SL printer platform, and de facto prevented the printing process. Therefore, 45 wt. % was chosen as the treated PEDOT:PSS percentage inside the PEGDA matrix to print the double helical devices.

### 3.3. Vapor Adsorption of PEGDA:PEDOT

#### 3.3.1. Electrical Characterization

**Figure 6:**
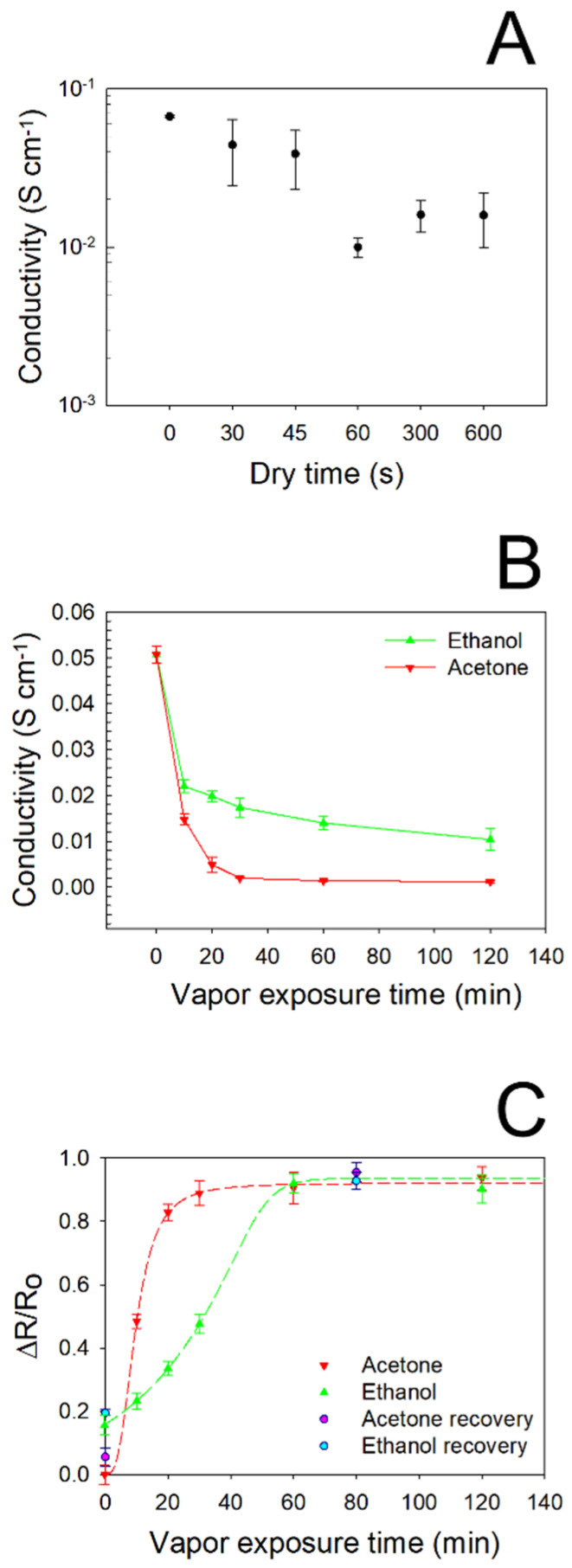
(**A**) Conductivity of PEGDA:PEDOT subjected to a 10 min acetone exposure after different drying times. (**B**) Conductivity of PEGDA:PEDOT for different exposure times to ethanol and acetone vapors. (**C**) Normalized resistance variation of the PEGDA:PEDOT samples versus acetone and ethanol exposure times.

In order to accurately assess the effect induced by vapor adsorption on the electrical features of PEGDA:PEDOT helical samples, the proper surface drying time was found by performing *I*/*V* measurements at controlled times after vapor exposure, by considering the maximum filler loading. In Figure 6A the typical σ values obtained for different drying times are reported for PEGDA:PEDOT submitted to a 10 min acetone exposure. Starting from the reference value of *σ* ≈ 0.055 S·cm^−1^, corresponding to the conductivity of the as-printed samples, we can observe that vapor adsorption induces a conductivity decrease. Nevertheless, drying times of 30 and 45 s appear to be not enough to induce a complete evaporation from the surfaces of the samples, as they provide non-reproducible conductivity measurements. On the other hand, a drying time of 300 s proves to be the minimum time necessary to obtain a good agreement between the results of *I*/*V* characterization. From this result, for all the samples the *I*/*V* and Raman spectroscopy characterizations were performed sequentially within 300 s after the drying process.

The vapors adsorption capacity of the PEGDA:PEDOT samples was studied by performing current–voltage measurements after the samples’ exposure to ethanol and acetone vapors for different times (Figure 6B). It can be seen from the graph that the conductivity of the samples significantly decreases only after a minimum exposure time of 10 min to both types of vapor. However, while acetone vapors cause a rapid decrease in conductivity with gradually increasing exposure times, the effect of ethanol is more moderate. In particular, it can be observed that after only 20 min of acetone exposure, the conductivity of the samples settles at a quasi-constant 10^−4^ S·cm^−1^ order of magnitude, varying from 13.7 × 10^−4^ to 9.1 × 10^−4^ S·cm^−1^ for 60 and 120 min, respectively. On the contrary, it takes about 60 min of ethanol exposure to reach a conductivity plateau of 13.5 × 10^−3^ S·cm^−1^. This result is well highlighted by the graph in Figure 6C, where the normalized resistance variation of the PEGDA:PEDOT samples versus acetone and ethanol vapor exposure times is reported. We note that the curve referring to acetone reaches saturation after about 20 min, while the curve referring to ethanol after 60 min. Moreover, the curves are characterized by a different initial trend that can help to rationalize the obtained results. Indeed, the general decrease in conductivity induced by vapor-exposure can be explained by the ability of the samples to accommodate the vapor molecules. As previously pointed out from the structural analysis, the polymer network is given by the interplay of PEGDA and treated PEDOT:PSS chains that can be altered by their interaction with vapor molecules. The latter, in fact, when penetrating the polymeric network, could cause a distancing of the treated PEDOT:PSS, thus destroying the conductive pathways they have formed in the material bulk (49) (50). However, this process appears to be slower with ethanol vapor, probably because the PEDOT:PSS was subjected to a treatment just in the ethanol solvent during the purification of the pristine material. Finally, a general reduction in the charge transport properties can probably also be attributed to the modification of the PEDOT chains’ conformation following interaction with the vapor molecules (51) (52).

The refresh capacity of the composite was evaluated by subjecting the material to a mild heat treatment of 70 °C for 12 h, at the end of which an electrical characterization was again performed. As it can be noted, after the heat treatment the ΔR/R_0_ values approach the normalized resistance variation values collected before acetone and ethanol exposure, confirming the recovery behavior of the produced material (Figure 6C).

**Figure 7:**
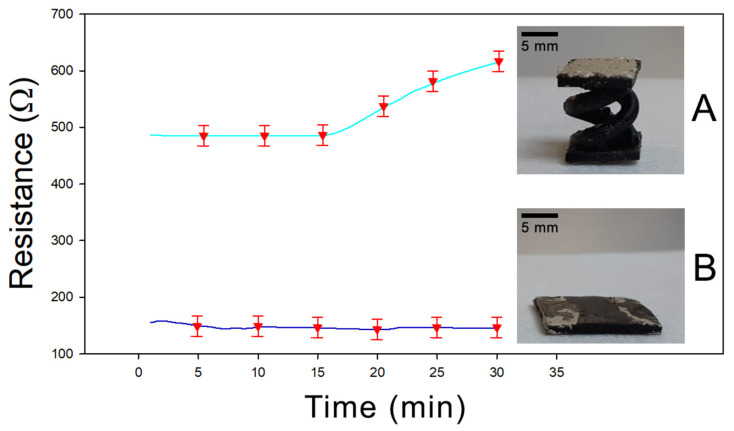
(**A**) Resistivity of 3D-printed double helical sample under acetone exposure. (**B**) Resistivity of 3D-printed parallelepiped shape under acetone exposure.

Finally, a further characterization was performed to evaluate the effect of the helicoidal geometry on the adsorption properties of the sample. In Figure 7, the resistance versus time diagram is reported for two different PEGDA:PEDOT samples having the same mass and composition but different shapes—a double helix and a rectangular parallelepiped with exposed surfaces of 5.4 cm^2^ and 5.7 cm^2^, respectively. In Figure 7A the response for a sample with a double helix structure is shown, while in Figure 7B the result for a sample with a rectangular parallelepiped shape is reported. It is evident how after 30 min of acetone exposure, the helical sample shows an increase in resistance about four times greater than the rectangular sample, confirming that the greater exposed surface related to the helical structure actually favors the vapor adsorption process in the final material.

#### 3.3.2. Raman Spectroscopy Analysis

**Figure 8:**
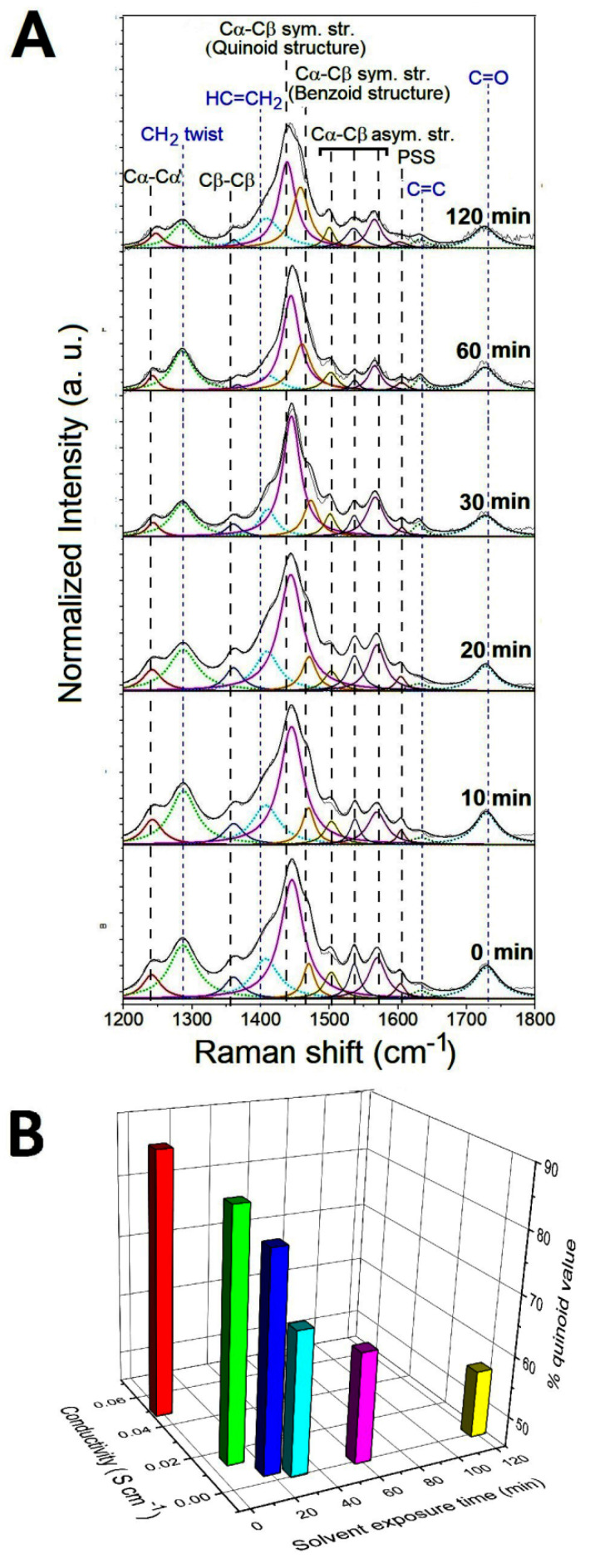
(**A**) Raman spectra of PEGDA:PEDOT samples exposed to acetone vapors for 0, 10, 20, 30, 60 and 120 min. (**B**) Conductivity and % quinoid value as a function of the vapor exposure time.

From the previous electrical characterization, it turns out that the response of the composite to ethanol is slower than the response to acetone. For this reason, only the conformational modifications of the PEDOT chains induced by the interaction with the acetone vapor molecules have been investigated in detail by analyzing the molecular structure of the PEGDA:PEDOT samples with Raman spectroscopy. In particular, the typical Raman spectra collected from the samples exposed to acetone vapors for 0, 10, 20, 30, 60 and 120 min are shown in Figure 8A. Considering that a drying time of 300 s allows for a reproducible electrical response, before the Raman characterization each sample was left to dry out for 5 min under environmental conditions.

As a general trend, one can observe that vapors produce no substantial changes in the Raman features of PEDGA chains. In fact, the signals related to this polymer phase are found to be significantly broader and less defined after only two hours exposure. This finding clearly suggests how PEGDA behaves as a robust support matrix for the whole composite. On the contrary, substantial variations in the spectral signatures of the PEDOT chains are found as a function of the exposure time to the acetone vapors. To quantify the extent of such modifications, the percentage contents of the extended quinoid molecular structures in PEDOT chains were determined by using Equation (2). % quinoid values of 88, 85, 80, 68, 63 and 56 were obtained after exposure for 0, 10, 20, 30, 60 and 120 min, respectively. The percentage decrease of the quinoid conformation in the samples shows how the interaction with the acetone molecules induces a conversion to the benzoid form of the PEDOT chains, as a probable consequence of a charge-transfer complex between the polar sites of the acetone molecules and the positively charged sites of the oxidized PEDOT chains. The increase in the benzenoid conformation leads to a general lower stacking of the polymer chains responsible for the worsening of the charge transport between these chains [41,42]. These considerations are well represented by the histogram graph in Figure 8B, where the conductivity and % quinoid values are both reported as a function of the vapor exposure time. The monotonic decrease in the two parameters confirms the strong correlation between the structural features of the PEDOT chains and the electrical properties of the whole PEGDA:PEDOT system.

## 4. Discussion

Given the performance exhibited by the PEGDA:PEDOT composite, a possible use as a cumulative measuring device for short-term or single-shot measurements is proposed [49,50]. In this view, in Table 1 a state-of-the-art review of the most relevant results collected for 3D-printed cumulative adsorption materials is shown to highlight the novelty of our achievements.

As can be observed from Table 1, a wide variety of materials have been printed by ink-jet technology, whereas MWCNT- and PEDOT:PSS-based composite materials have been essentially tested in fused deposition modeling and stereolithography printing production methods. Moreover, it is worth pointing out that the SL approach allows for printing high-surface area, complex architectures with a greater resolution compared to the ink-jet and FDM printing techniques.

As shown in Table 1, the normalized resistance variation of the double helix structure is consistent with the values reported in the literature. In particular, among the materials containing PEDOT:PSS filler, the printed PEGDA:PEDOT double helix structure exhibits the highest active area value and an ∆R/R ratio satisfactory for cumulative adsorption applications.

From this perspective, the coupling of the material and the printing technique adopted in the present work permits one to produce 3D structures with large and complex surface areas, that can be suitably exploited for the assembling of cumulative gas adsorption devices.

## 5. Conclusions

This work reports on the preparation of a new resin for stereolithographic 3D printing, based on a composite with PEGDA as the host matrix and PEDOT:PSS as the filler for 3D systems with VOC adsorbent properties. The control of the conductive features of PEDOT:PSS through suitable chemical treatments, and the identification of its appropriate loading in the host matrix, allowed the production of a homogeneous and easily printable resin for the manufacturing of conductive 3D objects with complex and reproducible geometries. These systems have proven able to interact with chemical vapors in the long-term by providing consistent and detectable variations in their structural and conductive characteristics.

The collected results demonstrate that the materials and the manufacture protocol here reported constitute a proof of principle of the possibility of developing an innovative manufacturing technology for the long-term monitoring of hazardous organic compounds based on cumulative adsorption effects.

## Figures and Tables

**Table 1:** State-of-the-art of 3D-printed cumulative adsorption materials for acetone and ethanol vapor. TPU: thermoplastic polyurethane polymer; PC: polycarbonate; PEDOT:PSS: poly(3,4-ethylenedioxythiophene) polystyrene sulfonate; PVDF: poly(vinylidene flouride); MWCNT: multi-walled carbon nanotubes; rGO: graphene oxide; CNFs: carbon nanofibers; FDM: fused deposition modeling; SL: stereolithography.

Printing Technique	Filler	Matrix	∆R/R	Active Area (mm^2^)	Ref.
Ink-jet	MWCNT + PEDOT:PSS	dH_2_O	0.02	1	[51]
Ink-jet	PEDOT:PSS	dH_2_O	0.03	40	[52]
Ink-jet	Graphene+PEDOT:PSS	dH_2_O	0.008	3.75	[53]
Ink-jet	CB	TPU	0.2	400	[54]
Ink-jet	rGO	dH_2_O	0.3	400	[55]
FDM	MWCNT	PC	3	8.13	[56]
FDM	MWCNT	PVDF	1.3	5.5	[57]
SL	CNFs	Epoxy resin	0.23	485	[58]
SL	treated PEDOT:PSS	PEGDA	1	540	This work

## Data Availability

Data sharing not applicable.
